# Duodenal Adenocarcinoma With Suspected Brain Metastasis

**DOI:** 10.7759/cureus.38199

**Published:** 2023-04-27

**Authors:** Sanchit Duhan, Bijeta Keisham, Chetna Duhan, Sahib Singh, Anubhav Jain

**Affiliations:** 1 Internal Medicine, Sinai Hospital of Baltimore, Baltimore, USA; 2 Radiodiagnosis, Smt Bhikhiben K Shah Medical Institute and Research Centre, Sumandeep Vidyapeeth, Vadodara, IND; 3 Cardiology, Ascension Genesys hospital, Grand Blanc, USA

**Keywords:** gastrointestinal adenocarcinoma, progressive dysphagia, brain met, small bowel malignancy, duodenal neoplasm

## Abstract

Duodenal adenocarcinoma (DA) is a rare tumor. We present the case of an 84-year-old lady who presented with episodic emesis with progressive dysphagia to solids and liquids. She also noted a significant weight loss of 31kg over four months. She was reported to have multiple brain masses three months before this admission. A computed tomography (CT) scan showed a heterogeneous mass (8cm) in the left retroperitoneum, inseparable from the duodenum. Additional peritoneal nodules and enlarged retroperitoneal lymph nodes were suspicious for metastases. Esophagogastroduodenoscopy revealed extrinsic compression of the stomach by the tumor. A large friable distal duodenal mass (fourth part) partially obstructed the lumen, which was biopsied. Pathology results demonstrated high-grade dysplasia but did not confirm malignancy. The patient's carcinoembryonic antigen (CEA) was elevated, but cancer antigens (CA)125 and CA19-9 were normal. A percutaneous biopsy of the mass revealed enteric-type adenocarcinoma. Immunohistochemistry showed that the tumor was positive for caudal-type homeobox (CDX)2, negative for special AT-rich sequence-binding protein (SATB)2, and patchy positive for cytokeratin (CK)7 and CK20 staining. The collective evidence suggested a duodenal primary. The patient opted for hospice and died in three days. We lack pathological evidence, but the patient's brain masses were suspicious of metastases. This would be one of the few reported cases of DA with possible brain metastases.

## Introduction

Small intestinal cancers are rare. The most frequently involved segment is the duodenum. Duodenal adenocarcinoma (DA) accounts for 0.5% of all gastrointestinal (GI) tumors [1]. The incidence of small bowel adenocarcinoma (SBA) has increased primarily secondary to an increase in DA [2,3]. Most DA patients are asymptomatic. They are diagnosed at an advanced stage during a routine medical checkup or surveillance esophagogastroduodenoscopy (EGD) and have a poor prognosis [4]. Some Japanese studies have reported that diagnosis at an early stage is associated with favorable outcomes [5,6]. However, given the rarity of the disease, the risk factors are unclear, making an early diagnosis difficult [7]. DA can metastasize to the liver and peritoneum, but brain metastasis is rare [8]. We present this rare case of DA with suspicious brain masses.

## Case presentation

An 84-year-old lady presented with lethargy and emesis for two days. She was experiencing dysphagia for two weeks, progressing from solids to liquids. It worsened to postprandial emesis with every meal. She also noticed an unintentional weight loss of 31 kg over four months. She denied any current or prior use of cigarettes and alcohol. Her family history was significant for breast cancer in her mother.

**Figure 1:**
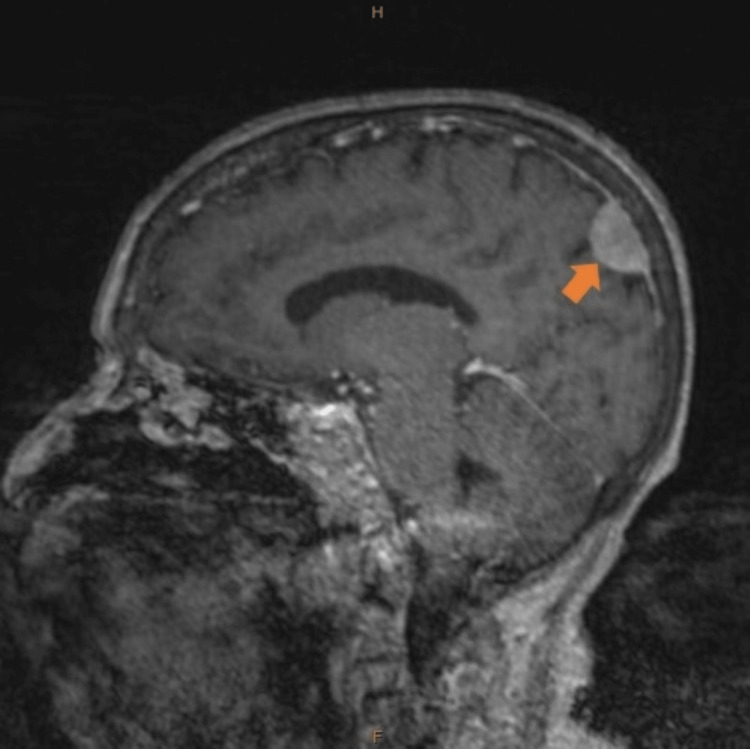
T1 sagittal magnetic resonance imaging (MRI) brain showing left parafalcine parietal lobe mass (arrow). H- head end, F- foot end.

**Figure 2:**
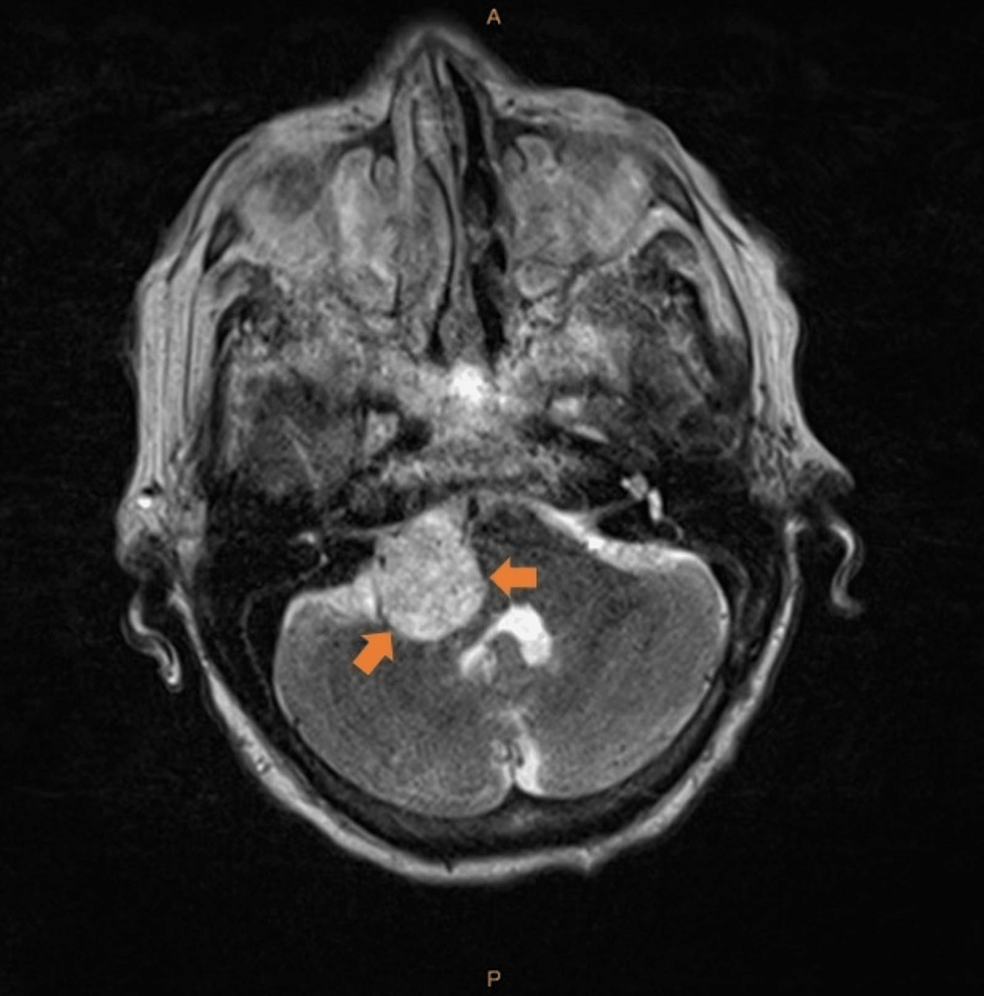
Axial T2 turbo spin-echo (TSE) magnetic resonance imaging (MRI) brain showing right cerebellopontine angle mass. A- anterior, P- posterior.

Three months before this admission, she had a stroke. Magnetic resonance imaging (MRI) brain revealed acute frontal and midbrain infarcts. Incidentally, several enhancing masses were seen throughout the supratentorial and infratentorial brain. A mass in the left parafalcine parietal lobe at the vertex measured 2 x 1.5 x 1.8 cm (Figure 1). A midline anterior cerebral mass measured 0.6 x 0.5 cm. Another mass was seen in the right temporal lobe along the dural surface of the sphenoid bone, measuring 0.6 x 1 cm. A mass in the right cerebellopontine angle measured 2.5 x 2.7 x 3 cm with intra-canalicular extension (Figure 2). These masses were new compared to a prior computed tomography (CT) scan obtained a year ago. The etiology of the masses was unclear, and the patient was advised to follow up outpatient. However, she was not able to attend her appointments.

**Figure 3:**
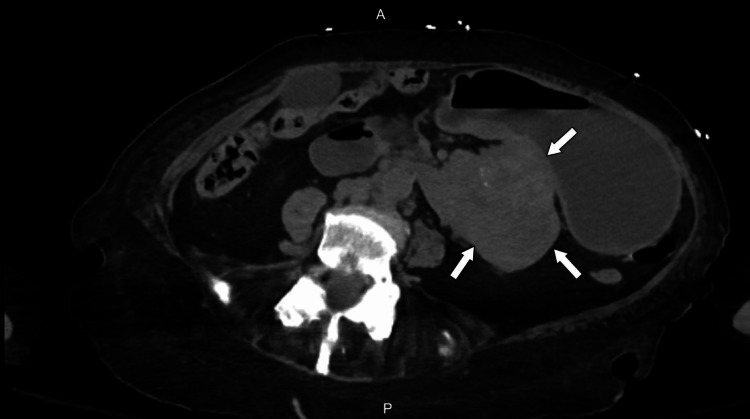
Abdominal computed tomography (CT) scan showing left retroperitoneal/duodenal mass (arrows). A-anterior, P-posterior.

On this admission, she had mild left upper quadrant tenderness but no palpable masses or distention on examination. Her overall presentation was concerning for a malignancy. A CT scan showed an 8 cm left retroperitoneal/duodenal mass and solid and cystic lobulated masses in the pelvis measuring up to 5 cm (Figure 3). Some hepatic and pulmonary nodules were also noted. Additional peritoneal nodules and enlarged duodenal, retroperitoneal, and paraesophageal lymph nodes were concerning for metastases. The differential diagnoses included a GI or genitourinary malignancy.

**Figure 4:**
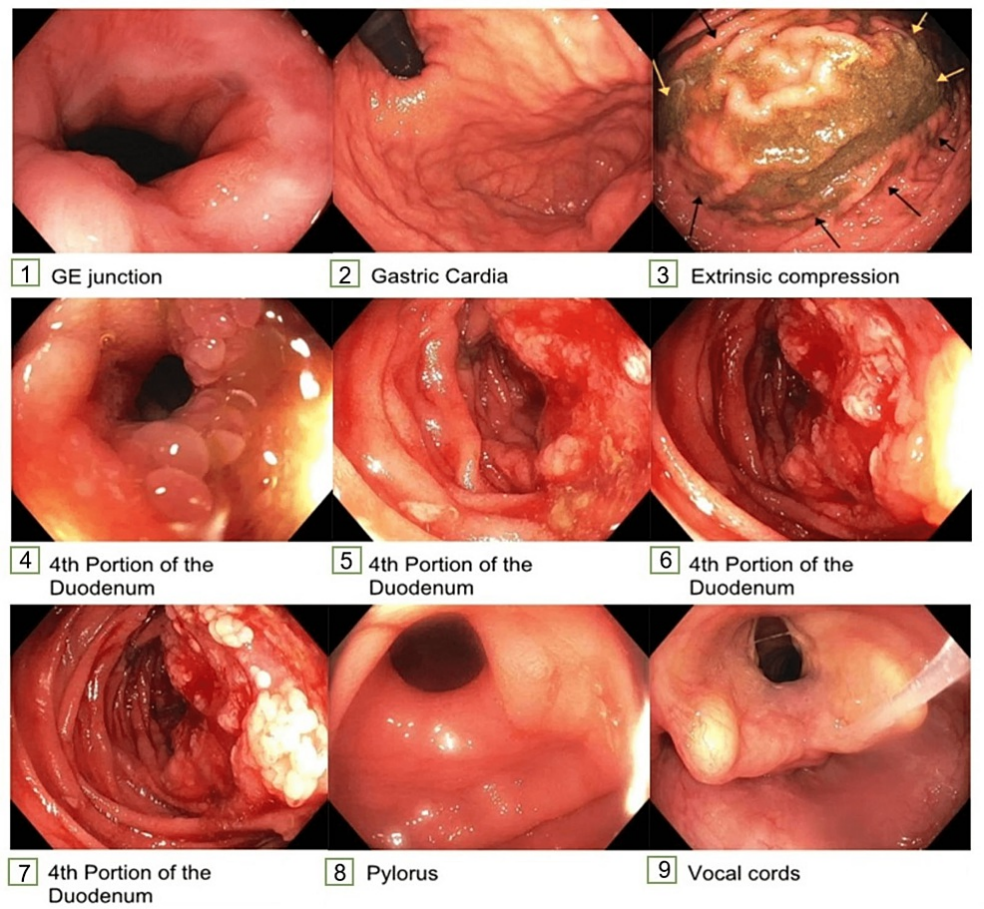
Esophagogastroduodenoscopy (EGD) images. Panel 1- normal gastroesophageal (GE) junction, panel 2- normal gastric cardia, panel 3- extrinsic compression of the greater curvature of the stomach, panel 4 to 7- villous, fungating, and friable mass in the fourth part of the duodenum, panel 8- normal pylorus, panel 9- normal vocal cords.

To obtain a biopsy, she underwent EGD, which showed extrinsic compression of the greater curvature of the stomach. A large fungating and friable mass in the duodenum's fourth part occupied 50% of the lumen. Retained gastric fluid suggested gastroparesis or small bowel obstruction (Figure 4). The duodenal mass was biopsied, which showed high-grade dysplasia and could not confirm the primary malignancy. Serum tumor markers were obtained to identify the primary source. Her serum carcinoembryonic antigen (CEA) and lactate dehydrogenase (LDH) were elevated. Other markers, including cancer antigen (CA) 19-9, CA-125, alpha-fetoprotein, and human chorionic gonadotropin, were within normal limits (Table 1).

**Table 1:** Tumor markers. CEA- carcinoembryonic antigen, LDH- lactate dehydrogenase, CA-cancer antigen, AFP- alpha-fetoprotein, hCG- human chorionic gonadotropin.

Tumor markers	Patient values	Reference range
CEA	783.8	0-5 ng/mL
LDH	629	84-246 U/L
CA 19-9	25.1	1.2-35 U/mL
CA-125	28.8	0-35 U/mL
AFP	< 2.0	0-8.8 ng/mL
hCG	3.0	0-5 mIU/mL

**Figure 5:**
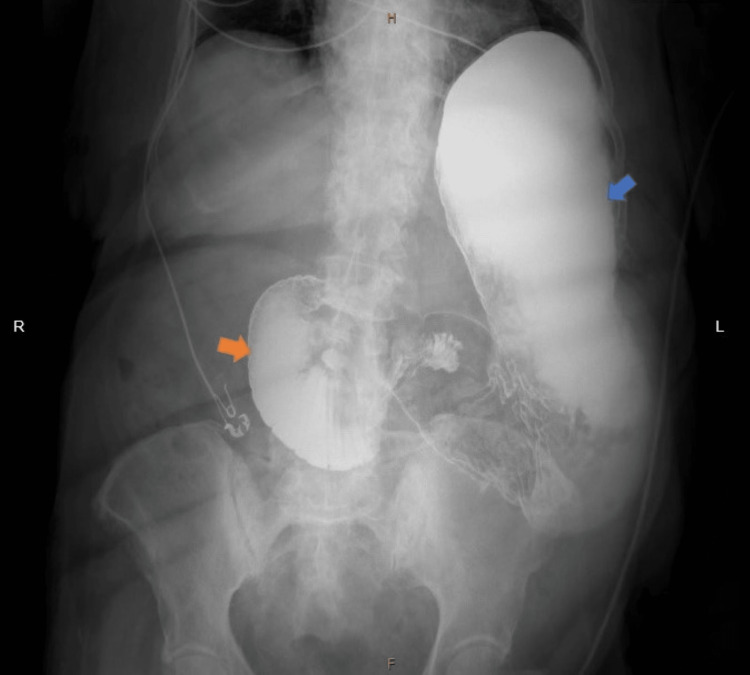
Upper gastrointestinal (GI) series showing markedly dilated stomach (blue arrow) and duodenum (orange arrow). R- right, H- head end, F- foot end, L- left.

Given the suspicion of bowel obstruction, she underwent an upper GI series. It showed a markedly dilated stomach and duodenum proximal to the previously seen duodenal/retroperitoneal mass on the CT scan (Figure 5). There was a significant delay in transit of the contrast to jejunal loops. This suggested a substantial narrowing of the duodenum's third part due to the tumor. A downstream complete small bowel obstruction could not be ruled out.

**Figure 6:**
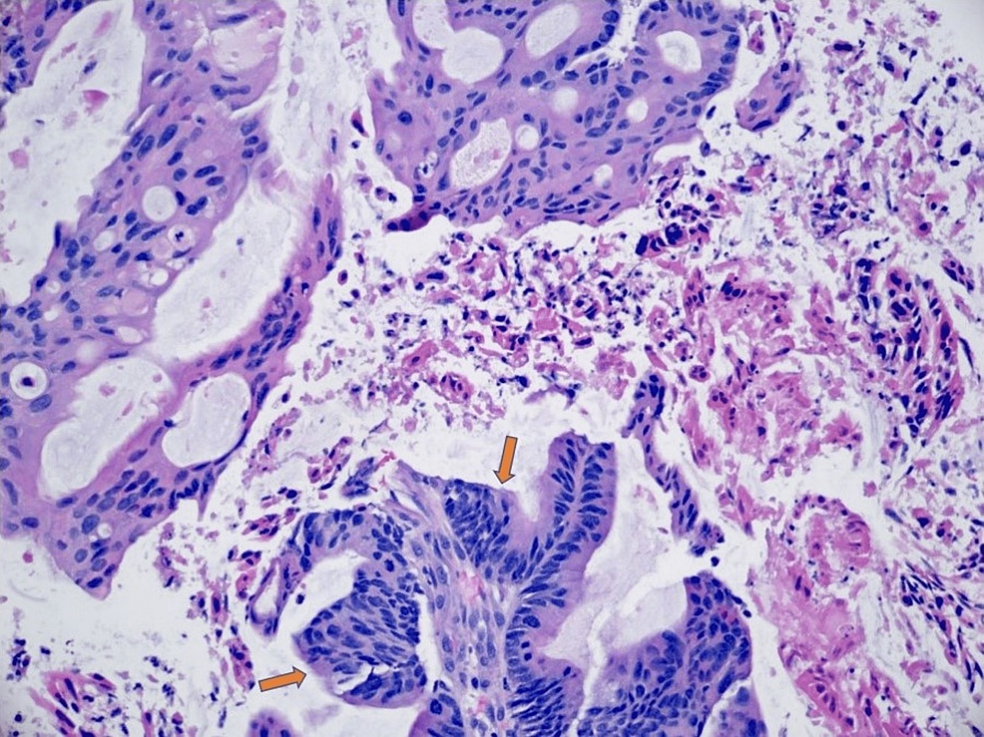
Carcinoma with gland formation (arrows) showing a gastrointestinal adenocarcinoma.

She continued to have daily episodes of emesis, and a nasogastric tube was placed for gut decompression. As the endoscopic biopsy was inconclusive, a percutaneous CT-guided biopsy of the retroperitoneal mass was obtained. Pathology reports confirmed GI adenocarcinoma (Figure 6). Immunohistochemistry (IHC) analysis showed that the sample was caudal-type homeobox 2 (CDX2) positive, patchy cytokeratin (CK) 7 and CK 20 positive, and special AT-rich sequence-binding protein (SATB) 2 negative. Since SATB2 was negative, a colonic primary was unlikely, and the IHC markers suggested DA as the primary malignancy. A diagnosis of DA with possible brain metastases was made.

Given the metastatic nature of the malignancy, the patient was not a candidate for surgery. She was offered a venting percutaneous gastrostomy for comfort, but the patient decided against it. She opted for comfort care measures and was discharged to inpatient hospice, where she died three days post-discharge.

## Discussion

There are rare reports of SBA with brain metastasis. One such case was reported by Yamazawa et al., who found a poorly differentiated SBA arising from the jejunum with a 38mm cystic metastasis in the left cerebellum [9]. Salvati et al. studied 10 cases of cerebral metastasis with SBA and noted most lesions to be supratentorial [10]. This case reports several small supra and infratentorial masses and a bigger right cerebellopontine angle mass. We do not have a pathological confirmation of the brain lesions. But given the identification of lesions only months before terminal decompensation of the patient with DA and no lesions seen on previous imaging, we suspect this is a case of metastatic brain lesions from the duodenal primary. 

Contrast-enhanced CT is weakly recommended for diagnosing distant metastases of DA. The evidence on the role of MRI is lacking [11]. We could identify a parafalcine lesion on the CT scan, but other metastases could be discovered only with an MRI. This report supports the use of MRI in determining distant metastases in DA.

The diagnosis of DA requires a detailed histopathologic examination of the tissue specimen. All the other GI sources of adenocarcinoma must be ruled out. Histologically, SBA is similar or indistinguishable from lower GI tract adenocarcinomas. However, they show essential epidemiological, clinical, and molecular differences from colorectal carcinomas (CRCs). These include 1) declining CRC incidence and rising SBA incidence, 2) worse prognosis of SBA patients than CRC patients with a five-year survival of 35% and 51.5 %, respectively, and 3) differences concerning the percentage of genetic driver mutations such as tumor protein (TP) 53 (58.4% in SBA vs. 75% in CRC), adenomatous polyposis coli (APC) (26.8% vs. 75.9%) and cyclin-dependent kinase Inhibitor (CDKN) 2A (14.5% vs. 2.6%), leading to potential therapeutic implications [12-14]. The IHC profile of lower GI tract (colorectal or appendiceal) carcinomas is CK7 negative/CK20 positive/CDX2 positive. On the other hand, 60% of SBAs co-express CK7 and CK20, and 50% are negative for CDX2. SATB2 expression is restricted to glandular cells lining the lower GI tract (appendix, colon, and rectum). It has been recently proposed as a tissue-specific and sensitive marker of lower GI tract adenocarcinomas. However, the studies on SATB2 expression in SBA are limited [15]. In this case, the anatomical location of the mass on imaging and endoscopy, along with IHC markers, indicated the diagnosis as metastatic DA.

The association of CA 19-9 and CEA is not well established in DA. However, recent data suggest that an elevation of both markers is associated with worse overall survival and higher prognostic impact of CA 19-9 than CEA [16]. In this case, CEA was elevated, CA 19-9 was normal, and the patient had a rapid malignancy progression. More prospective trials are needed to validate the associations of these markers with DA.

The treatment options for DA comprise surgical, endoscopic, chemoradiation, and immunotherapy approaches. The lymph node metastases occur when DA extends deeper than the submucosal layer. There is no evidence showing the prognostic effect of lymph node dissection. Peripheral lymph node dissection may be considered if it can be safely resected [11]. Pancreato-duodenectomy is the currently proposed standard procedure for DA in the submucosal layer or deeper. However, no efficacy and safety of the procedure is established [11]. Surgical gastric jejunal bypass and endoscopic stent insertion are believed to improve quality of life and restore oral intake. However, no evidence to support this is sufficient, and these procedures have weak recommendations. Different retrospective studies and meta-analyses have shown varying evidence for perioperative adjuvant chemotherapy/radiotherapy/chemoradiotherapy. The current consensus weakly recommends against postoperative adjuvant chemotherapy [11]. Systemic chemotherapy with pyrimidine fluoride and oxaliplatin is recommended as primary therapy for unresectable or recurrent DA. Microsatellite instability (MSI) testing is strongly recommended for unresectable or recurrent DA. Among immune checkpoint inhibitors, pembrolizumab is strongly recommended for previously treated unresectable or recurrent DA with MSI-high or deoxyribonucleic acid (DNA) mismatch repair deficiency (dMMR) [11]. However, our patient declined any interventions. It is beyond the scope of this report to comment on these treatment modalities.

## Conclusions

This is a rare case of DA with possible brain metastasis. The primary was identified using histopathological, radiological, and endoscopic techniques. The IHC panel was used to differentiate it from CRC. DA is difficult to diagnose early, and the treatment options are still in developmental stages due to the tumor's rarity.
